# The Overlap of Posterior Reversible Encephalopathy Syndrome and Reversible Cerebral Vasoconstriction Syndrome: A Report of a Complex Case and Challenges With Its Management

**DOI:** 10.7759/cureus.88916

**Published:** 2025-07-28

**Authors:** Ana Tojal, Elisabete Monteiro

**Affiliations:** 1 Intensive Care Medicine Department, Unidade Local de Saúde Gaia/Espinho, Vila Nova de Gaia, PRT; 2 Intensive Care Medicine Department, Centro Hospitalar Universitário de São João, Porto, PRT

**Keywords:** cerebral vasospasm, cerebral vasospasm, hypertensive encephalopathy, posterior reversible encephalopathy syndrome (pres), reversible cerebral vasoconstriction syndrome (rcvs), serotonin syndrome, vasogenic brain edema

## Abstract

We report the case of a 38-year-old female presenting with posterior reversible encephalopathy syndrome (PRES) overlapping with reversible cerebral vasoconstriction syndrome (RCVS), diagnosed based on clinical and imaging criteria. The patient exhibited progressive neurological symptoms, including headache, visual disturbances, and motor deficits. Brain magnetic resonance angiography revealed vasogenic edema and vascular stenoses. The complications included severe agitation, sympathetic hyperreactivity, and features suggestive of serotonin syndrome. The management focused on blood pressure control, symptom management, and medication adjustment. Despite significant improvement during hospitalization and rehabilitation, residual deficits persisted. This report highlights the complex interplay between PRES and RCVS, emphasizing the importance of serial neuroimaging, systematic medication reviews, and a multidisciplinary approach in managing such overlapping conditions.

## Introduction

Posterior reversible encephalopathy syndrome (PRES) is a neurotoxic condition characterized by a failure in autoregulation of the posterior cerebral circulation, often precipitated by acute changes in blood pressure. The syndrome is thought to result from a complex interplay of factors, including endothelial dysfunction, failure of cerebral autoregulation, and immune-mediated processes. Acute hypertension is believed to overwhelm the cerebrovascular autoregulatory capacity, causing hyperperfusion, blood-brain barrier disruption, and subsequent vasogenic edema. Conversely, a subset of patients without severe hypertension may develop PRES due to other triggers, such as sepsis, cytotoxic medications, fluid overload, or renal failure, highlighting the multifactorial nature of its pathogenesis [1-4].

The clinical manifestations of PRES typically encompass a spectrum of symptoms, including headache, seizures, visual disturbances, altered mental status, and focal neurological deficits. Diagnosis is primarily radiological, with brain MRI being the most useful diagnostic tool. Characteristic findings include bilateral vasogenic edema, most commonly affecting the parieto-occipital regions; however, atypical presentations involving the frontal lobes, cerebellum, or brainstem have also been reported [1-3,5]. While the syndrome was initially considered entirely reversible, studies have shown that incomplete recovery or recurrent episodes can occur, particularly if the underlying causes are not promptly addressed [3,5]. However, its prevalence is not known.

There is a relevant overlap between PRES and reversible cerebral vasoconstriction syndrome (RCVS) in terms of clinical presentation, imaging, and pathophysiology [6]. PRES is primarily associated with hyperperfusion due to autoregulatory failure, while RCVS is characterized by severe vasospasm and hypoperfusion. Recent studies suggest that shared mechanisms such as endothelial dysfunction, blood-brain barrier breakdown, and sympathetic hyperactivity may underlie both conditions [7]. Clinically, RCVS often presents with recurrent thunderclap headaches and may be triggered by exposure to vasoactive substances (e.g., serotonergic agents, sympathomimetics), postpartum state, or physical exertion. Studies suggest that PRES may progress to RCVS due to vascular injury and reactive vasospasm, whereas approximately 9-38% of RCVS cases may lead to PRES through mechanisms such as acute hypertension and endothelial dysfunction [8]. This relationship highlights the need for an individualized approach to diagnosis and management. This case report aims to highlight the diagnostic and therapeutic challenges in managing overlapping features of PRES and RCVS.

## Case presentation

Initial presentation

The patient was a 38-year-old female with a history of chronic obstructive pulmonary disease (COPD) classified as GOLD A, arterial hypertension without known target organ damage, depressive syndrome, and substance abuse, including cannabis. Her regular medications included mexazolam (1mg/day), trazodone (150mg/day), bupropion (150mg/day), and fluoxetine (20mg/day).

**Figure 1:**
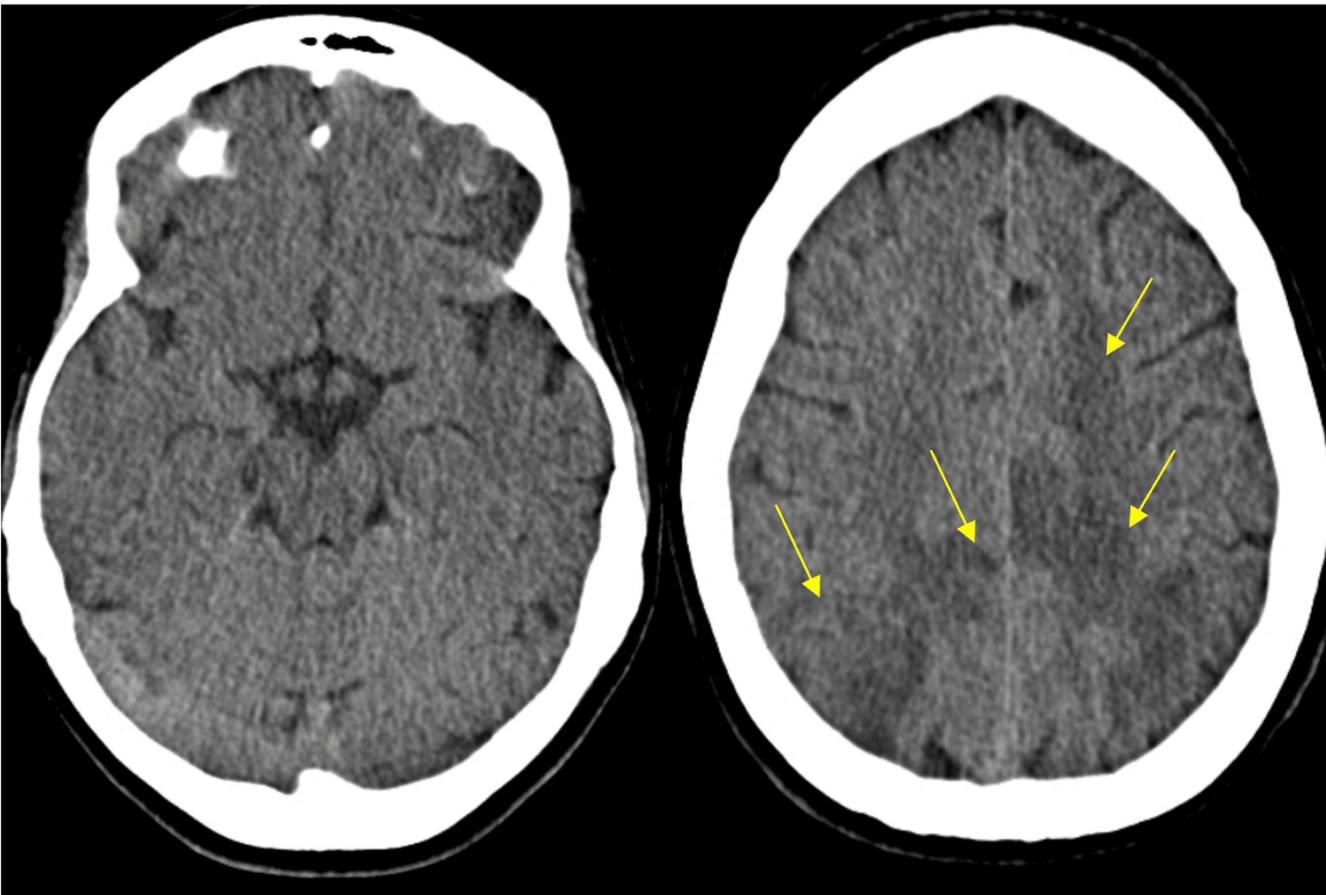
Emergency department cranial CT Hypodense areas involving the white matter bilaterally in high fronto-parietal subcortical planes, and subcortical and cortico-subcortical areas of the bilateral parieto-occipital region (yellow arrows), suggestive of vasogenic edema CT: computed tomography

The patient presented with a two-week history of nausea, vomiting, and headache, followed by a 12-hour progression of worsening headache and visual disturbances. Neurological examination in the emergency department revealed paraparesis of the lower limbs, right upper limb paresis, right central facial paresis, bilateral inexhaustible ankle clonus, and bilateral extensor plantar reflexes. A cranial CT scan suggested PRES/RCVS overlap (Figure 1). 

**Figure 2:**
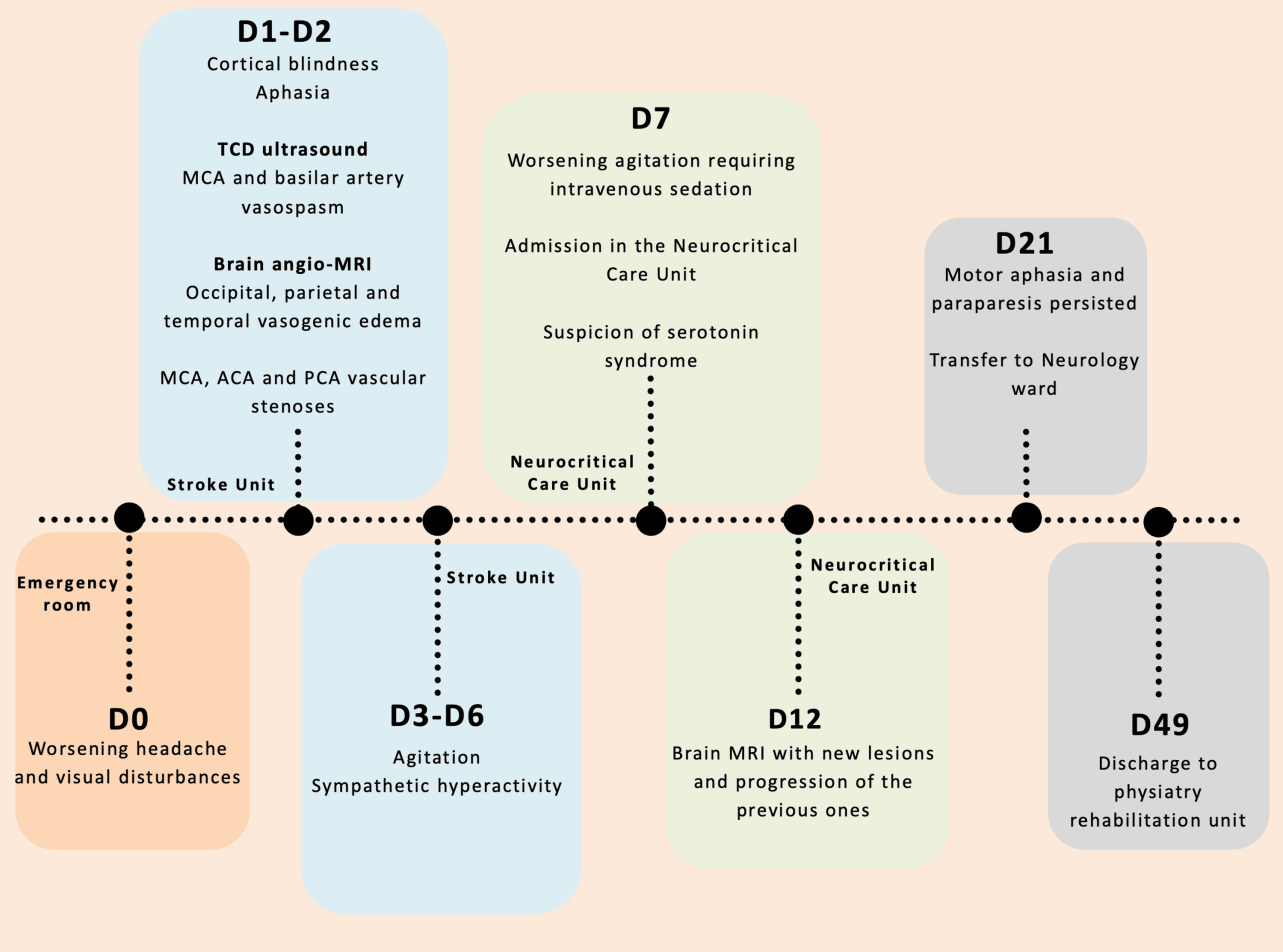
Chronological timeline summarizing key events ACA: anterior cerebral artery; MCA: middle cerebral artery; MRI: magnetic resonance imaging; PCA: posterior cerebral artery; TCD: transcranial Doppler

Urine toxicology was positive for cannabinoids and benzodiazepines. A chronological timeline summarizing the key events is depicted in Figure 2.

Treatment course in the stroke unit

**Figure 3:**
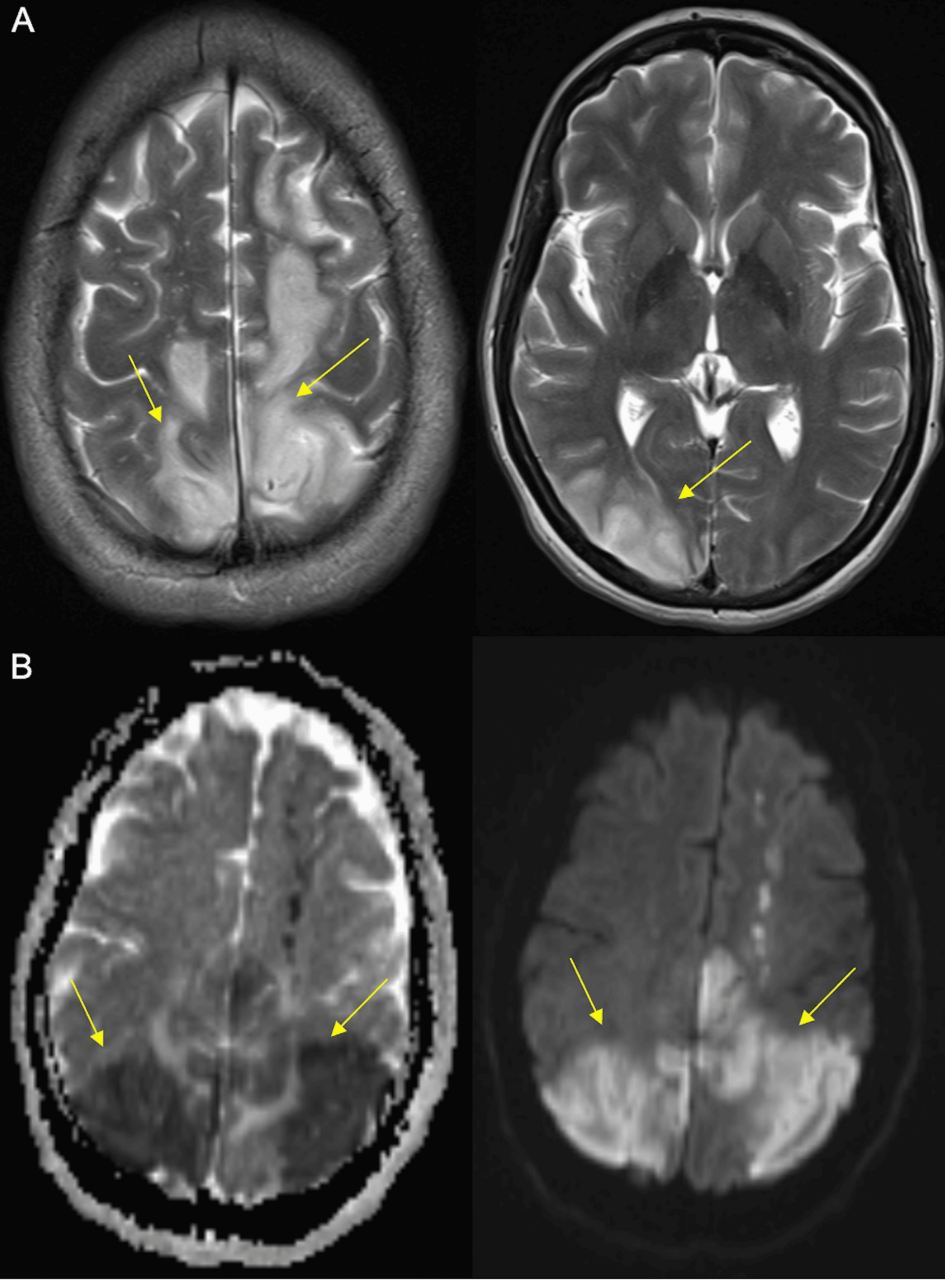
Brain MRI at admission Panel A: axial T2-weighted images. Panel B: axial ADC image on the left and diffusion-weighted image on the right side (yellow arrows pointing to areas of cytotoxic edema) ADC: apparent diffusion coefficient; MRI: magnetic resonance imaging

The patient was admitted to the stroke unit, where her condition deteriorated, with the presence of severe headaches, cortical blindness, aphasia, and agitation, requiring intravenous sedation. Transcranial Doppler ultrasound revealed moderate vasospasm in the left middle cerebral artery (MCA) and mild vasospasm in the right MCA and basilar artery. Brain angio-MRI demonstrated cortico-subcortical vasogenic edema in the right occipito-parieto-temporal region, bilateral parieto-occipital regions, centrum semiovale on the left, and a right posterior subinsular focus (Figure 3).

Multiple vascular stenoses were identified in the MCA, anterior cerebral artery (ACA), posterior cerebral artery (PCA), and basilar arteries.

Neurocritical care course

On the seventh day of hospital stay, the patient developed a clinical picture of sympathetic hyperreactivity: diaphoresis, tachycardia, hypertension, bilateral mydriasis, hyperreflexia, inexhaustible clonus, bilateral Babinski sign, and muscular rigidity. She was transferred to the neurocritical care unit at this point, where she continued to exhibit significant agitation, requiring multiple oral and intravenous medications, initially including continuous infusions of dexmedetomidine and remifentanil and an intravenous bolus of midazolam. The suspicion of serotonin syndrome was raised, given the use of trazodone, bupropion, and fluoxetine as her usual medication and the use of additional perpetuating medications during hospitalization, including remifentanil, buspirone, and sertraline, which were discontinued at this stage (on the third day of stay in the neurocritical care unit). 

Supportive laboratory workup, including creatine kinase, bicarbonate, and metabolic panel, was performed as part of the differential diagnoses and to monitor potential complications, but yielded no specific abnormalities. However, it was difficult to determine whether all symptoms could be exclusively attributed to serotonin syndrome or if some might be, at least partially, explained by withdrawal phenomena (the patient had history of substance abuse and it was not entirely clear if she had active consumption at the time of hospital admission) or cerebral vasospasm (of which there was no further evidence on transcranial doppler the day following neurocritical unit admission). Agitation, along with consequent hypertension and tachycardia, may have been exacerbated by the patient’s partial awareness of her neurological deficits, although this remains a clinical impression. Additionally, the hypothesis of pheochromocytoma as a potential cause of the sympathetic hyperactivity was considered, but it was excluded after analytical and imaging evaluations.

**Figure 4:**
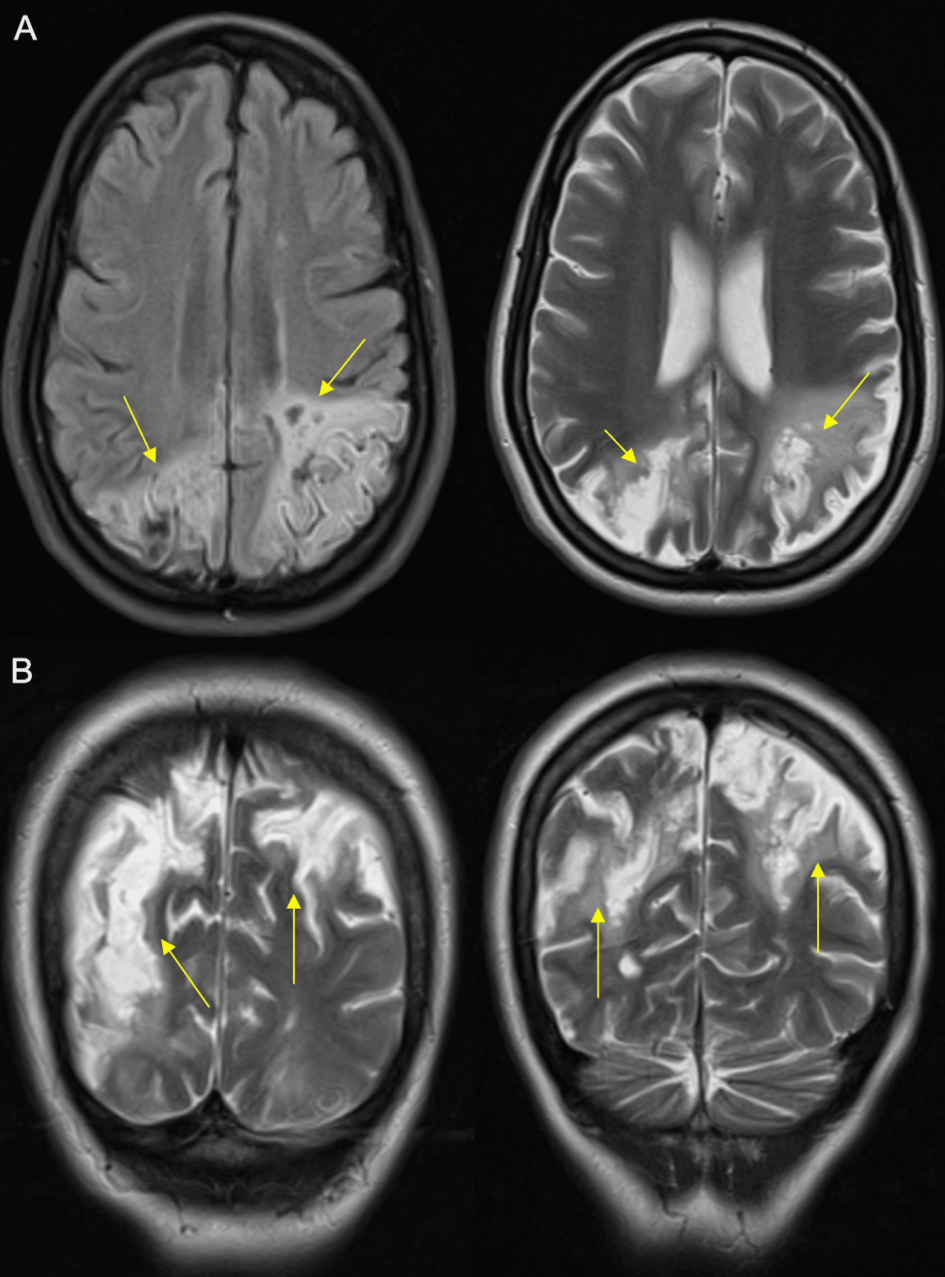
Brain MRI - revaluation T2-weighted images. Panel A: axial plane. Panel B: coronal plane. Yellow arrows pointing to the lesion areas MRI: magnetic resonance imaging

A 48-hour period of orotracheal intubation was required to achieve deeper sedation for a new brain MRI (Figure 4) on the 12th day of hospital stay, which showed progression of existing lesions and new lesions in the left occipital cortex, right thalamopeduncular region, cingulate gyrus, and medial aspect of the left superior frontal gyrus. 

Given the persistence of severe agitation and neurological deficits, a brain biopsy was discussed with the Neurosurgery team. However, the patient's condition evolved favorably, and dexmedetomidine and intravenous midazolam were successfully discontinued, and the brain biopsy was no longer considered.

Throughout hospitalization, the patient experienced hypertensive periods that appeared to correlate with moments of intensified agitation. Initially, during her stay in the stroke unit, hypertension was managed with nimodipine. Following transfer to the neurocritical care unit, the patient’s blood pressure management required escalation to short-acting agents such as urapidil and labetalol due to significant blood pressure lability. This tensional lability is characteristic of PRES but may also have been influenced by the continuous infusion of sedative medications administered to manage agitation. Once clinical stability was achieved, the patient was transitioned to propranolol and clonidine, which provided effective and sustained blood pressure control. 

Recovery phase

At the time of discharge from the neurocritical care unit to the neurology ward, the patient was on alprazolam, baclofen, and oral midazolam for anxiety and agitation control and propranolol and clonidine for hypertension management. At this stage, the patient exhibited motor aphasia, being able to express herself with simple words despite significant echolalia. She followed commands, presented with right-sided hemiplegia and left-sided hemiparesis graded at 3+/5, and showed apparent cortical blindness. 

During her stay in the neurology ward, verbal communication improved, although mixed aphasia persisted. In this phase, the patient was completely dependent on others for all basic activities of daily living. She was then transferred to a physiatry rehabilitation unit for continued recovery, where, after two months, she showed partial recovery from cortical blindness and left hemiparesis, with improvement in speech and comprehension. However, right-sided hemiplegia and bilateral inexhaustible ankle clonus persisted.

## Discussion

This report exemplifies the complexities of diagnosing and managing the overlap between PRES and RCVS in a multifactorial context. The coexistence of these conditions involves a dynamic interaction of pathophysiological mechanisms, including endothelial dysfunction, autoregulatory failure, and reactive vasospasm. Additional mechanisms, such as sympathetic overactivity and reperfusion injury, have also been proposed and may contribute to the overlapping clinical and radiological features. These theories support the concept of a shared spectrum rather than distinct entities in some cases [6,7]. The patient's presentation aligns with known risk factors for PRES, such as severe hypertension and substance use [5,9], which likely played a central role in the pathogenesis. Radiological findings were crucial in confirming the diagnosis, emphasizing the significance of brain MRI in detecting bilateral vasogenic edema and vascular stenoses. 

This report also highlights the importance of considering PRES in patients with atypical presentations. While the classic description of PRES involves predominantly posterior brain regions, our patient exhibited more extensive involvement, including frontal and thalamic regions. This is consistent with literature suggesting that atypical patterns of PRES are more common than previously thought [7,10,11]. The progression of imaging abnormalities over time further underlines the need for repeat evaluations to guide management [10]. The overlap of PRES and RCVS is particularly noteworthy. It has been reported that approximately 9-38% of RCVS cases may lead to PRES, underlining the interrelated nature of these conditions [5,12]. Our case supports this observation and underscores the need for clinicians to be vigilant for signs of both syndromes when either is suspected. It also supports the hypothesis that PRES and RCVS exist on a spectrum of cerebrovascular dysregulation rather than as completely distinct entities [9].

The suspected serotonin syndrome episode in this patient added a layer of complexity, illustrating how medication interactions can exacerbate neurological dysfunction in the context of overlapping syndromes. While features such as severe agitation, hyperreflexia, clonus, and autonomic instability were consistent with serotonin syndrome, differential diagnoses, including withdrawal phenomena and dysautonomia secondary to PRES/RCVS, were also considered. The diagnosis was supported by clinical application of the Hunter Serotonin Toxicity Criteria [13], and careful exclusion of alternative causes. This case emphasizes the importance of a systematic medication review and cautious use of serotonergic agents in patients with polypharmacy or psychiatric comorbidities. Furthermore, this underlines the importance of managing contributing factors, including hypertensive crises and the discontinuation of serotonergic agents [14].

Therapeutic strategies in this case focused on strict blood pressure control, symptomatic management, and supportive care, reflecting current best practices for PRES [5,7]. The use of calcium channel blockers may have mitigated the risk of worsening vasospasm, consistent with the overlap between PRES and RCVS. The difficult hypertension control also highlights the challenges of managing hypertension in PRES, particularly in patients with concomitant agitation and sedation requirements. The partial neurological recovery observed accentuates the potential reversibility of PRES when promptly and adequately treated; however, the persistence of deficits, such as motor aphasia and hemiparesis, indicates the importance of early rehabilitation and long-term follow-up. 

PRES is a condition that can significantly impact patients' quality of life, and currently, no therapy has been definitively shown to alter patient outcomes [2]. Further research is needed to identify reliable biomarkers or imaging features that enable early differentiation between PRES and RCVS, as well as to establish optimal blood pressure management strategies in cases where both syndromes coexist.

## Conclusions

This report contributes to the existing literature on PRES and RCVS by highlighting the diagnostic and management complexities involved, underscoring the importance of a multidisciplinary approach. It highlights several key points: firstly, the overlap between PRES and RCVS is more common than previously recognized, necessitating a high index of suspicion for both conditions when either is present. Secondly, atypical radiological presentations of PRES are frequent and should not dissuade clinicians from the diagnosis when clinical features are suggestive. Thirdly, while PRES is often considered reversible, a significant proportion of patients may have persistent neurological deficits, emphasizing the need for early aggressive management and long-term follow-up. The report also underscores the complexity of managing these patients, particularly when complicating factors such as serotonin syndrome are present. It demonstrates the importance of careful medication management and the need for vigilant monitoring of blood pressure and neurological status. A better understanding of these overlapping syndromes will be crucial for advancing patient care and reducing long-term morbidity. Future research efforts should include the development of standardized treatment protocols, investigation of potential biomarkers for early diagnosis and prognosis, and exploration of targeted therapies that may improve outcomes in both PRES and RCVS. Gaining a better understanding of the pathophysiology underlying these conditions will improve our ability to provide more effective and personalized care for affected patients.
